# Paternal smoking and preterm birth: a population-based retrospective cohort study among non-smoking women aged 20–49 years in rural China

**DOI:** 10.1186/s12978-022-01378-x

**Published:** 2022-03-24

**Authors:** Long Wang, Yuzhi Deng, Ying Yang, Fangchao Liu, Qin Xu, Zuoqi Peng, Yuan He, Yuanyuan Wang, Jihong Xu, Hongguang Zhang, Ya Zhang, Qiaomei Wang, Haiping Shen, Yiping Zhang, Donghai Yan, Xu Ma

**Affiliations:** 1 National Research Institute for Family Planning, No. 12, Dahuisi Road, Haidian, Beijing, 100081 China; 2 Graduate School of Peking, Union Medical College, No. 9 Dong Dan San Tiao, Dongcheng, Beijing, 100005 China; 3 Institute of Epidemiology and Statistics, School of Public Health, Lanzhou University, Lanzhou, 730000 Gansu China; 4 National Human Genetic Resources Center, No. 12, Dahuisi Road, Haidian, Beijing, 100081 China; 5 Department of Epidemiology, Fuwai Hospital, National Center for Cardiovascular Diseases, Chinese Academy of Medical Sciences and Peking Union Medical College, No. 167 Beilishi Road, Xicheng, Beijing, 100037 China; 6 Department of Maternal and Child Health, National Health and Family Planning Commission of the PRC, No. 1, Xizhimenwai Road (South), Xicheng, Beijing, 100044 China

**Keywords:** Tobacco smoking, Paternal smoking, Preconception, Preterm birth

## Abstract

**Background:**

To comprehensively evaluate the association of paternal smoking and preterm birth (PTB).

**Methods:**

We performed a population-based retrospective cohort study in rural areas of China among 5,298,043 reproductive-aged couples who participated in the National Free Pre-Pregnancy Checkups Project (NFPCP), regarding outcome events that occurred in 2010–2016. Multivariate Cox proportional regression was used to estimate hazard ratio (HR) and 95% confident intervals (95%CI), and restricted cubic spline (RCS) were used to estimate the dose–response relationship.

**Results:**

Compared to neither-smoker couples, the fully adjusted HR for PTB was 1.04 (95% CI, 1.03–1.04), 1.08 (0.96–1.22), and 1.11 (1.03–1.19) in the couples where only the female smoked, only the male smoked and both, respectively. HR of PTB for paternal smoking was 1.07 (1.06–1.07), compared with women without paternal smoking. Consistent with paternal smoking, preconception paternal smoking showed 1.07-fold higher risk of PTB (95%CI, 1.06–1.09). The multivariable-adjusted HRs of PTB were 1.05 (1.03–1.06), 1.04 (1.03–1.05), 1.05 (1.04–1.07), 1.07 (1.05–1.10) and 1.13 (1.12–1.14) for participants whose husband smoked 1–4, 5–9, 10–14, 15–19, and ≥ 20 cigarettes/day respectively, compared with participants without paternal smoking. The HRs of PTB also increased with the increment of paternal smoking and preconception paternal smoking categories (***P***_linear_ < 0.05).

**Conclusions:**

Paternal smoking and preconception paternal smoking was independently positively associated with PTB risk. The importance of tobacco control, should be emphasized during preconception and pregnancy counselling should be towards not only women but also their husband.

**Supplementary Information:**

The online version contains supplementary material available at 10.1186/s12978-022-01378-x.

## Plain language summary

Preterm birth (PTB) is the leading cause of neonatal morbidity and mortality, as well as other system immaturity problem. But as one of important environmental tobacco smoking sources of maternal secondhand smoking in the family, paternal smoking is worthy for deep exploring of its potential impacts on PTB. Moreover, evidence on the independent role of preconception paternal smoking is still lacking. Thus, we conducted a population-based retrospective cohort study to evaluate the association between paternal smoking and risk of PTB among among 5,298,043 reproductive-aged couples who participated in the National Free Pre-Pregnancy Checkups Project (NFPCP), regarding outcome events that occurred in 2010–2016.

We found a significant association between paternal smoking and PTB. Smoking reduction should not only be advised to pregnant women but also to their partners to reduce PTB in their fetal. In addition, supporting patients to continue smoking reduction will be crucial when considering the adverse health outcome of smoking. Intervention of tobacco use before and during pregnancy, are critical for prevention of PTB. Avoiding both maternal and paternal smoking during pregnancy will benefit the developing fetus.

**Supplementary Information:**

The online version contains supplementary material available at 10.1186/s12978-022-01378-x.

## Background

Preterm birth (PTB), defined as delivery before 37 completed weeks, is the leading cause of neonatal morbidity and mortality, as well as other system immaturity problems like neurodevelopmental, pulmonary, gastrointestinal, immunological or cardiovascular issues [1, 2]. Multiple factors could contribute to the rising risk of PTB, including preterm ruptured membranes, spontaneous preterm labor, or twins or high-order multifetal pregnancies [2, 3]. Maternal cigarette smoking and passive smoking are both established risk factors for PTB [4]. But as one of the important environmental tobacco smoking sources of maternal secondhand smoking in the family, paternal smoking is worthy of deep exploring of its potential impacts on PTB. Moreover, evidence on the independent role of preconception paternal smoking is still lacking.

World widely, males were almost five times more likely to use tobacco compared with females [5]. In the Americas and European countries, the prevalence of smoking in males is 18% higher than that in females; while in China, the ratio between male smokers and female smokers reached 30 times (55.7% vs 1.9%) among people aged 18–49 years [6]. The separate and combined effects of maternal and paternal smoking on the offspring during the different stages of pregnancy should also be noticed, and more comprehensive research is needed to provide solid evidence for the relationship between paternal smoking and PTB.

Thus, we conducted a population-based retrospective cohort study to evaluate the association between paternal smoking and risk of PTB among over 5 million non-smoking women aged 20–49 years in rural China based on National Free Pre-pregnancy Checkups Projects (NFPCP).

## Material and methods

### Study participants and study design

The NFPCP is a national project providing free preconception health examination and counseling reproductive-health services for rural couples, supported by the National Health and Family Planning Commission and Ministry of Finance of China since 2010. Detailed design, implementation and published articles of this program can be found elsewhere [7–9], and a flow figure for design of the NFPCP was included in Additional file 2: Fig. S1.

Briefly, Couples planning to conceive within the next 6 months were encouraged to participate in NFPCP. After the initial preconception health examination, participants were followed up by telephone for every 3 months within one year after the examination to obtain conception status by health care professionals from local family planning service agencies or maternal and child service centers until conception is confirmed. Participants who became pregnant were asked to come back to the service agencies/centers to undergo ultrasonography and have a physician diagnosis to confirm the pregnancy about 2 months after the last menstrual period (LMP). Once pregnancy was confirmed, pregnant participants were re-followed up for the pregnancy outcome information within one year.

This study was approved by the Institutional Research Review Board at the National Health and Family Planning Commission, now known as National Health Commission.

### Data collection

At health examination, participants’ demographical characteristics were collected using standard and structured questionnaire, including lifestyle information, history of adverse pregnancy outcomes and disease history. The physical and clinical data were also obtained. Conception status, LMP, smoking habits for both couples were collected at early pregnancy follow-up interview. During early pregnancy, women reported pregnant were asked to come back to the clinic to undergo ultrasonic examinations and have a physician’s diagnosis to confirm the pregnancy about 2 months after the LMP, and the first day of the LMP was adjusted by ultrasonic examinations this time. In the final stage, the participants who had become pregnant were recontacted for pregnancy outcome information within 1 year after the completion of the first follow-up survey. Written informed consents were obtained from all NFPCP participants.

### Exposure, outcome and covariates

Paternal smoking was set as the primary exposure in this study. “Did you smoke? If yes, how many cigarettes were smoked in one day?” was asked. Cigarette smoking was defined as participants with at least 1 cigarette/day at preconception health examination. Paternal smoking was categorized as 0, 1–4, 5–9, 10–14, 15–19, and ≥ 20 cigarettes/day. According to the smoking status of couples, we classified participants into four groups: (1) neither maternal nor paternal smoking (neither-smoker), (2) only maternal smoking (maternal-only), (3) only paternal smoking (paternal-only), and (4) both smoking (both smokers). Periconception paternal smoking was defined as an exposure of cigarettes before pregnancy, thus participants whose husband still smoked during early-pregnancy or did not provide such information were excluded in periconception paternal smoking.

The main outcome is preterm birth, defined as delivery before 37 completed gestational weeks.

Confounders used in model adjustment include maternal and paternal age at LMP, higher education, Han Chinese ethnic, preconception body mass index (BMI), alcohol drinking, parental passive smoking, parity, history of adverse pregnancy outcomes, and region of service station.

Definitions of the covariates are simply described here. Age was defined as exact years between birthdays and LMP. Higher education was defined as levels of education of senior high school, or higher. History of adverse pregnancy outcome was defined as a history of adverse events in the previous pregnancies. Preconception BMI was calculated as kilograms per square meters (kg/m^2^). Parity was categorized as primiparity and multiparity. Region of service station was classified into 7 groups according to location of provinces, including Northeast, North, Northwest, East, South, and Southeast.

All missing values of categorical variables were recoded as a new category.

### Statistical analysis

We used Cox proportional regression to estimate the adjusted hazard ratios (HRs) and its 95% confidence intervals of parental smoking and paternal smoking on PTB. Tests for the linear trend of ORs were conducted by modelling the median value of each paternal smoking category in a simple linear regression. We also examined the dose–response relationship of paternal smoking and PTB by restricted cubic spline (RCS), and the knots for the RCS were set at the 5th, 35th, 65th, and 95th percentiles of dosage. The non-linearity of dose–response was tested by Wald statistics [10].

For each outcome, three Cox proportional hazards regression models were fitted. Model I was adjusted for maternal age at LMP. Model II was adjusted for region of service station and baseline characteristics, including maternal and paternal age at LMP, higher education, Han Chinese ethnic, BMI, alcohol drinking, and parental passive smoking. Model III was additionally adjusted for TSH (Thyroid Stimulating Hormone), diabetes, hypertension, parity, and history of adverse pregnancy outcomes. Only the fully adjusted models were presented. Results for all models were given in the supplementary material.

Subgroup analyses on association between paternal smoking and PTB were conducted on parts of baseline characteristics. In addition, we also assessed whether the association between paternal smoking and PTB varied by subgroup characteristics through a test for interaction, whereas ***P***_multiplicativity_ was the ***P***-values for the product term of paternal smoking and subgroup characteristics.

To stress more on paternal smoking before conception, we also conducted association analyses after excluding participants whose husband still smoked or did not provide such information at early-pregnancy follow-up. And we defined participants with paternal smoking but quitted at early-pregnancy follow-up as preconception paternal smoking.

The R software (version 3.2.2, https://www.r-project.org/) was used for data analyses. For the linear trend test of ORs and the nonlinear test of RCS, a 2-sided ***P*** value < 0.05 was considered statistically significant. For interaction test for subgroup characteristics and paternal smoking on PTB, *P-*value < 0.25 was considered statistically significant.

## Results

**Fig. 1:**
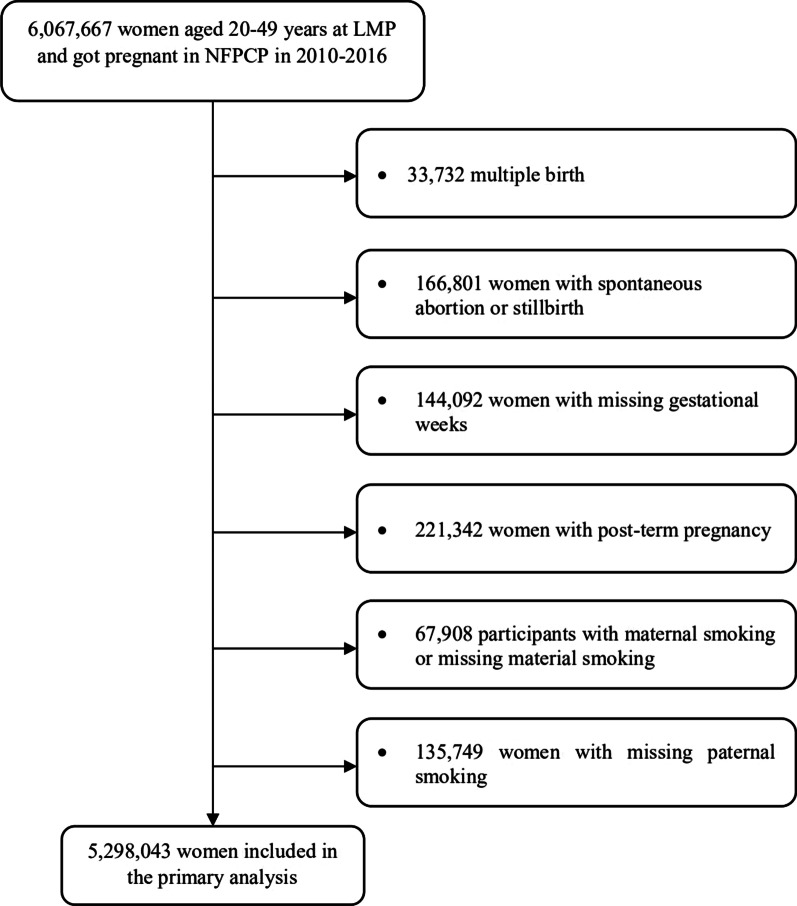
Flowchart of study population

The current study was based on NFPCP participants with outcomes that occurred in 2010–2016. A total of 6,067,667 participants aged 20–49 years at LMP were included; 33,732 participants with multiple births, 166,801 participants with spontaneous abortion or stillbirth, 144,092 participants with missing gestational weeks, 221,342 post-term pregnancies, 67,908 participants with maternal smoking or missing maternal smoking, 135,749 participants with missing paternal smoking were excluded sequentially. As result, 5,298,043 were included in primary analysis (Fig. 1). Baseline comparison between included and excluded participants is given in Additional file 1: Table S1.

Baseline characteristics of participants, according to paternal smoking status, showed that women with paternal smoking were more likely to be less educated, minority nationalities, multiparity, have history of adverse pregnancy outcomes, and more likely to have parental passive smoking (Table 1). Among women with paternal smoking, they had the average gestational age of 39 weeks and birth weight of 3.30 kg, at the meanwhile the women without paternal smoking had the average gestational age of 39 weeks and birth weight of 3.30 kg.

**Table 1:** Demographic characteristics of female participants by paternal smoking

Characteristics	Without paternal smoking (N = 3,760,013)	Paternal smoking (N = 1,538,030)	P-value
Maternal age at LMP, years			< 0.001
20–24	1,854,321 (49.32)	787,071 (51.17)	
25–29	1,435,650 (38.18)	552,020 (35.89)	
20–34	370,544 (9.85)	155,818 (10.13)	
34–39	84,127 (2.24)	36,769 (2.39)	
≥ 40	15,371 (0.41)	6352 (0.41)	
Paternal age at LMP, years			0.698
20–24	1,187,331 (31.58)	513,454 (33.38)	
25–29	1,723,283 (45.83)	663,335 (43.13)	
20–34	608,613 (16.19)	251,416 (16.35)	
34–39	175,640 (4.67)	80,289 (5.22)	
≥ 40	51,510 (1.37)	23,516 (1.53)	
NA	13,636 (0.36)	6020 (0.39)	
Higher education, N (%)	478,692 (12.73)	169,000 (10.99)	< 0.001
Han Chinese ethnic, N (%)	3,466,467 (92.19)	1,396,953 (90.83)	< 0.001
BMI		ef	0.015
Underweight	505,479 (13.44)	249,029 (16.19)	
Normal weight	2,750,542,(73.15)	1,058,090 (68.80)	
Over weight	401,370 (10.67)	184,711 (12.01)	
Obese	81,635 (2.17)	42,358 (2.75)	
NA	20,987 (0.56)	3842 (0.25)	
Alcohol drinking, ml/day			< 0.001
No	3,682,327 (97.93)	1,479,682 (96.21)	
1–10	7943 (0.21)	7113 (0.46)	
11–50	6172 (0.16)	5652 (0.37)	
≥ 51	2535 (0.07)	2531 (0.16)	
NA	61,036 (1.62)	43,052 (2.80)	
Primipara, No. (%)	2,655,253 (70.62)	1,010,155 (65.68)	< 0.001
History of adverse pregnancy outcome, N (%)	93,903 (2.50)	67,638 (4.40)	< 0.001
Maternal passive smoking, min/day			< 0.001
No	3,677,977 (97.82)	1,377,704 (89.58)	
1–15	52,305 (1.39)	97,893 (6.36)	
≥ 16	23,515 (0.63)	59,690 (3.88)	
NA	6216 (0.17)	2743 (0.18)	
Paternal passive smoking, min/day			< 0.001
No	3,619,027 (96.25)	1,173,620 (76.31)	
1–15	80,040 (2.13)	165,487 (10.76)	
≥ 16	52,868 (1.41)	192,236 (12.50)	
NA	8078 (0.21)	6687 (0.43)	

*BMI* body mass index, *LMP* last menstrual period, *TSH* thyroid stimulating hormone

Before excluding participants with maternal smoking or missing such information, we first investigated the association between parental smoking status and PTB. Compared neither-smoker couples, the fully adjusted HR for PTB was 1.04 (95% CI, 1.03–1.04), 1.08 (95% CI, 0.96–1.22), and 1.11 (95% CI, 1.03–1.19) in the paternal-only, maternal-only, and both smokers, respectively, which implied a significant adverse effect of paternal smoking on PTB (Table 2). Magnitude of the association between maternal smoking on PTB was stronger than paternal smoking did.

**Table 2:** Adjusted odds ratio of parental smoking status for preterm birth

Smoking amounts	No. (%)	OR (95% CI)		
		Model I^a^	Model II^b^	Model III^c^
Neither-smoker	3,760,013 (7.46)	1 [Reference]	1 [Reference]	1 [Reference]
Paternal-only	1,538,030 (8.12)	1.09 (1.08–1.10)	1.05 (1.04–1.06)	1.04 (1.03–1.04)
Maternal-only	3357 (8.25)	1.10 (0.98–1.23)	1.09 (0.96–1.22)	1.08 (0.96–1.22)
Mixed	8281 (8.57)	1.14 (1.06–1.23)	1.11 (1.03–1.20)	1.11 (1.03–1.19)

This analysis was conducted before excluding participants with maternal smoking or missing such information

^a^ORs were adjusted by maternal age at last menstrual period

^b^ORs were adjusted by maternal and paternal age at last menstrual period, maternal higher education, Han ethnic, preconception body mass index and alcohol drinking

^c^ORs were adjusted by paternal and maternal age at last menstrual period, maternal higher education, Han ethnic, body mass index, alcohol drinking, thyroid stimulating hormone, diabetes, hypertension, parity, history of adverse pregnancy outcomes, and region of service station

**Fig. 2:**
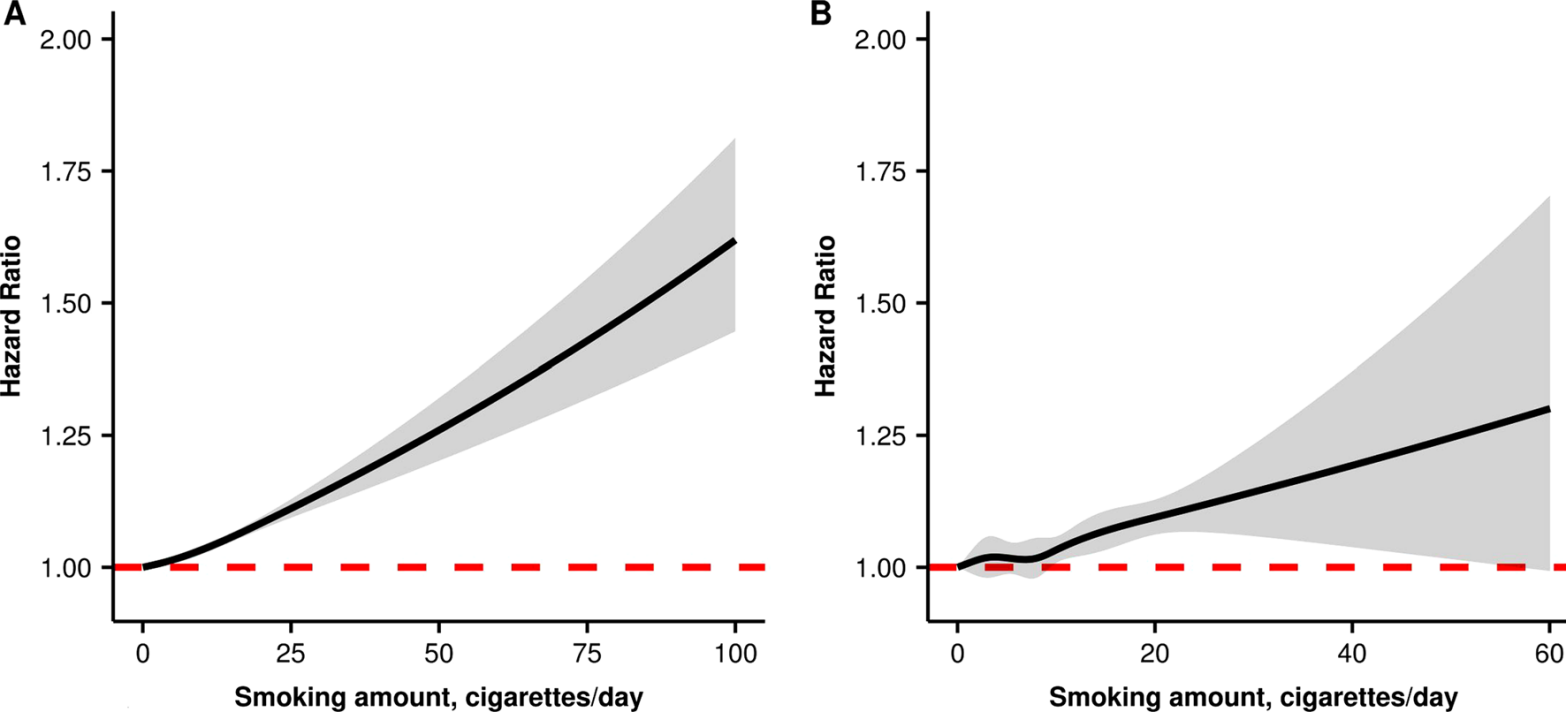
Dose–response relationship between paternal smoking and preterm birth. **A** paternal smoking; **B** preconception paternal smoking. The black curves represent adjusted odds ratios for paternal smoking based on restricted cubic splines with knots at the 5th, 35th, 65th, and 95th percentiles of smoking amount. Grey lines show the 95% confidence intervals of odds ratio for restricted cubic splines of paternal smoking (Parts of the lines were clipped for better comparison). Covariates used in the analysis included region of province, maternal age at last menstrual period, paternal age at last menstrual period, higher education, living area, Han ethnic, body mass index, alcohol drinking, parental passive smoking, thyroids stimulating hormones, diabetes, hypertension, parity, and history of adverse pregnancy outcomes. Red dashed lines demonstrate the reference level

**Fig. 3:**
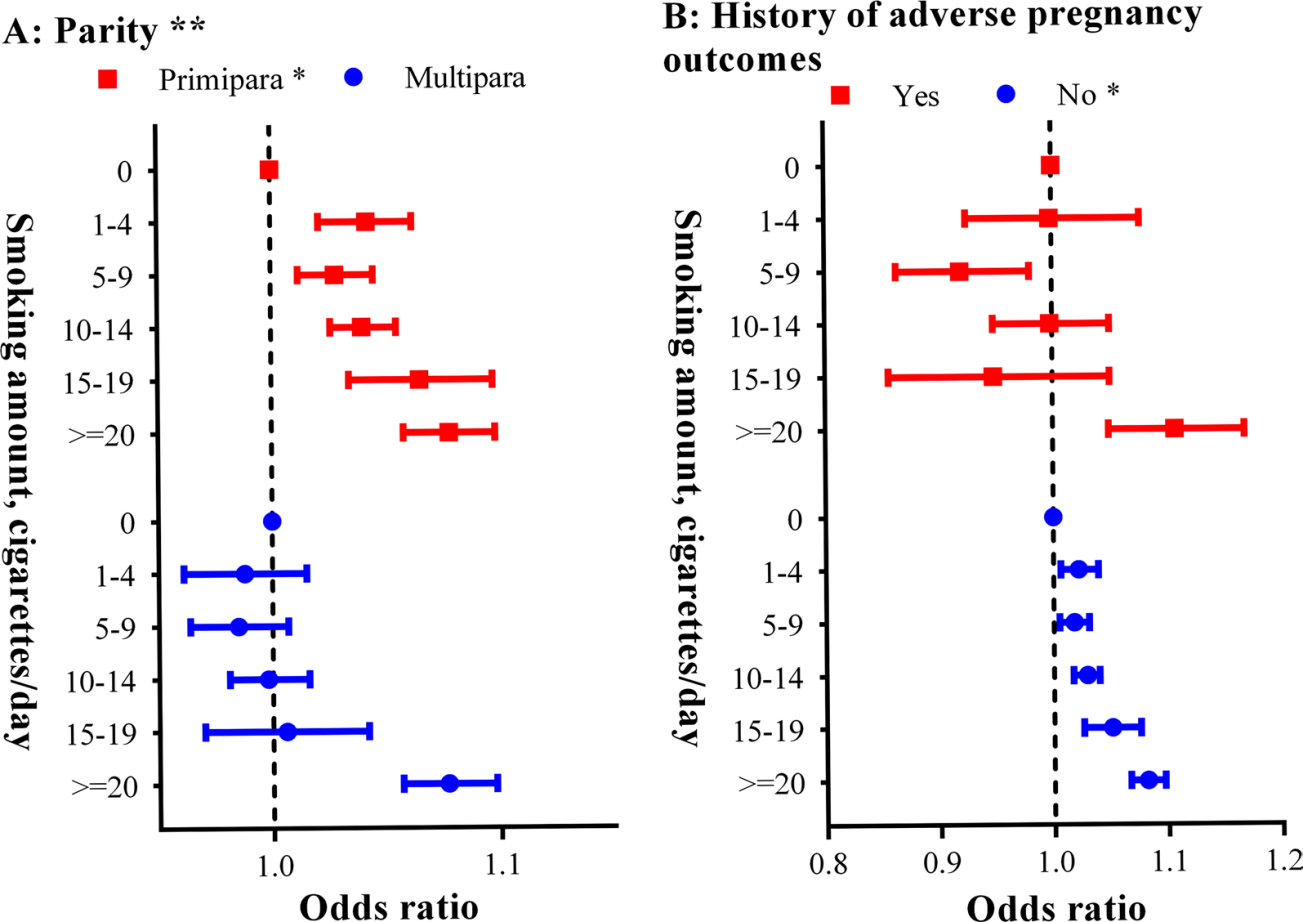
Subgroup analyses on association between paternal smoking and preterm birth. **A** parity; **B** history of adverse pregnancy outcomes. Point and bar represent odds ratio and its 95% confidence interval of paternal smoking and spontaneous abortion in fully adjusted logistic model. “*” stands for P linear < 0.05; “**” stands for P_multiplicativity_ < 0.25

In the fully adjusted model, paternal smoking was found to be associated with PTB (Table 3). HRs of PTB were 1.07 (1.06–1.07), compared with women without paternal smoking. Compared with participants without paternal smoking, the multivariable-adjusted HRs of PTB were 1.05 (95% CI,1.03–1.06), 1.04 (95% CI,1.03–1.05), 1.05 (95% CI,1.04–1.07), 1.07 (95% CI,1.05–1.10) and 1.13 (95% CI,1.12–1.14) for participants whose husband smoked 1–4, 5–9, 10–14, 15–19, and ≥ 20 cigarettes/day respectively. Paternal smoking was positively associated with PTB (***P***_linear_ < 0.05). As showed in Fig. 2A, the RCS result also revealed similar trend (***P***_nonlinear_ > 0.05). Subgroup analyses showed the results consisted on parties and history of adverse pregnancy outcomes (Fig. 3). But the associations varied across subgroup characteristics (***P***_multiplicativity_ < 0.25).

**Table 3:** Relationship between paternal smoking and preterm birth

Smoking amounts	No. (%)	HR (95% CI)	
		Model I^b^	Model II^c^
Paternal smoking			
No	3,760,013 (7.46)	1 [Reference]	1 [Reference]
Yes	1,538,030 (8.12)	1.09 (1.08–1.10)	1.07 (1.06–1.07)
1–4	208,098 (8.26)	1.11 (1.09–1.12)	1.05 (1.03–1.06)
5–9	360,518 (7.99)	1.07 (1.06–1.09)	1.04 (1.03–1.05)
10–14	537,788 (8.00)	1.07 (1.06–1.09)	1.05 (1.04–1.07)
15–19	101,598 (7.97)	1.07 (1.05–1.09)	1.07 (1.05–1.10)
≥ 20	317,154 (8.45)	1.14 (1.12–1.15)	1.13 (1.12–1.14)
*P *_*linear*_	–	0.140	0.007
Preconception paternal smoking^a^			
No	3,760,013 (7.46)	1 [Reference]	1 [Reference]
Yes	190,529 (8.12)	1.08 (1.06–1.10)	1.07 (1.06–1.09)
1–4	25,601 (8.16)	1.09 (1.04–1.13)	1.04 (0.99–1.08)
5–9	43,887 (8.02)	1.07 (1.04–1.11)	1.05 (1.01–1.08)
10–14	66,285 (7.91)	1.05 (1.02–1.08)	1.06 (1.03–1.09)
15–19	12,020 (8.34)	1.12 (1.05–1.19)	1.14 (1.07–1.21)
≥ 20	40,882 (8.37)	1.12 (1.08–1.16)	1.15 (1.11–1.19)
*P *_*linear*_	–	0.054	0.003

*CI* confidence interval, *OR* odds ratio

^a^Participants whose husband still smoked during early-pregnancy or did not provide such information were excluded

^b^ORs were adjusted by maternal age at last menstrual period

^c^ORs were adjusted by maternal and paternal age at last menstrual period, maternal higher education, Han ethnic, preconception body mass index and alcohol drinking

### Preconception paternal smoking and preterm birth

**Fig. 4:**
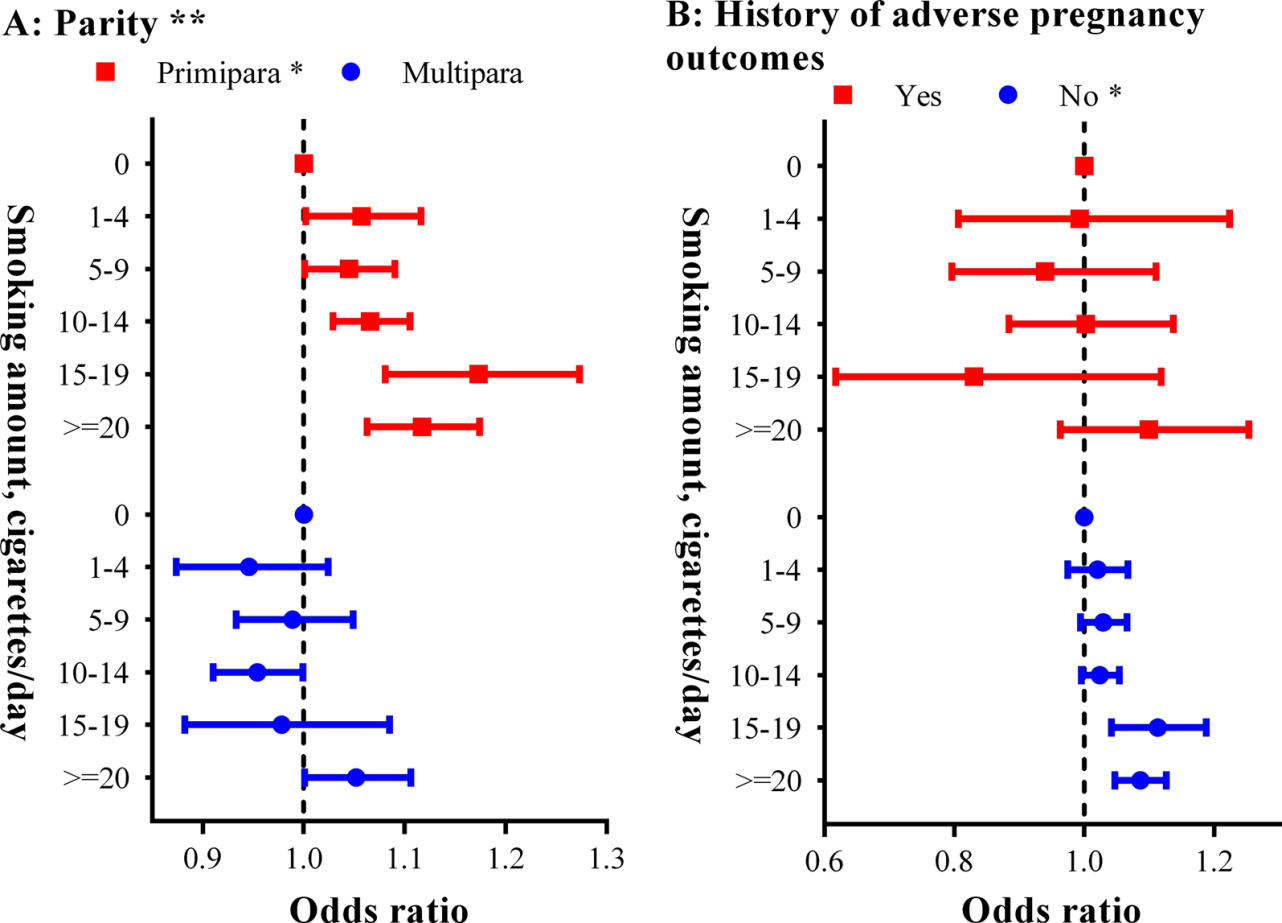
Subgroup analyses on association between preconception paternal smoking and preterm birth. **A** parity; **B** history of adverse pregnancy outcomes. Point and bar represent odds ratio and its 95% confidence interval of paternal smoking and spontaneous abortion in fully adjusted logistic model. “*” stands for P linear < 0.05; “**” stands for P _multiplicativity_ < 0.25

For preconception paternal smoking, our study showed consistent results as paternal smoking did on PTB risk. HR of PTB was 1.07 (95% CI, 1.06–1.09), compared with women without preconception paternal smoking. Compared with participants without preconception paternal smoking, the fully adjusted HRs of PTB were 1.04 (95% CI, 0.99–1.08), 1.05 ((95% CI, 1.01–1.08), 1.06 ((95% CI, 1.03–1.09), 1.14 ((95% CI, 1.07–1.21) and 1.15 ((95% CI, 1.11–1.19) for participants whose husband smoked 1–4, 5–9, 10–14, 15–19, and ≥ 20 cigarettes/day respectively (***P***_linear_ < 0.05). The RCS result also confirmed similar linear trend (***P***_nonlinear_ > 0.05). (Fig. 2B). Subgroup analysis showed the results consisted on parties and history of adverse pregnancy outcomes (**Fig. ****4**). But the associations varied across parties (***P***_multiplicativity_ < 0.25).

## Discussion

In this large-scale population-based retrospective cohort study, we found a significant association between paternal smoking and PTB. This study also provided considerable evidence on that preconception paternal smoking could independently increase the risk of preterm birth, which highlighted the importance of tobacco control of paternal smoking, and husband should quit smoking when planning a pregnancy to reduce PTB risks as much as possible.

Although maternal smoking and PTB have been frequently debated in pregnancy studies, few studies have considered the relationship between paternal smoking and PTB. Among previous studies using measures of paternal tobacco exposures, most of them obtained negative results, which were inconsistent with us [11–13]. A cohort study of 21,248 postpartum women conducted by Ting-Jung has failed to find a significant association of paternal smoking with preterm birth infants [11]. Another national prospective longitudinal cohort study in Indonesia also reported that there was not significant association between paternal smoking and PTB in both urban and rural areas [14]. There are two possible explanation, one is that the average amount of cigarettes smoked per day of husbands were not high enough to observe an effect on adverse pregnancy outcomes in their offsprings, and the other might be that they ignored the confounding effect of maternal passive smoking. Additionally, since the magnitude of association between paternal smoking and PTB was relatively weak, further potential impacts of paternal smoking on PTB subtype (e.g. very preterm birth) were not discussed here.

In our study, compared with non-smoking parents, both maternal and paternal smoking were significantly correlated with a higher risk of preterm delivery. The combining effect of maternal and paternal smoking on PTB was stronger than paternal smoking alone. These current results were consistent with studies that also reported significant increases in PTB related to parental smoking [14]. Thus, these facts could persuade couples who plan for pregnancy to quit smoking prior to pregnancy. Both males and females should know that exposure to prenatal tobacco smoking is associated with different adverse outcomes such as PTB.

The pathophysiology of the association between maternal smoking and PTB is described and supported in several previous studies. Nicotine can interfere with hypoxic embryogenesis, placentation and organogenesis during pregnancy by generating excess reactive oxygen species, which results in direct oxidative damage to the nuclear deoxyribonucleic acid (DNA) [15]. It could cause abnormal remodeling of the spiral arteries supplying the placenta and reduce nutrition and oxygen transfer required for proper fetal growth [15, 16]. Dysregulated functional and programming capacity of the placental-fetal unit is likely to influence PTB [17–21]. Unlike maternal smoking, paternal smoking involves distinct mechanisms on pregnancy. Paternal smoking could have an intrauterine effect on the fetus through passive smoking. Nicotine and carbon monoxide in the blood of a pregnant woman exposed to second-hand smoke can decrease the blood flow and oxygen in fetal [22]. Tobacco could also induce aneuploidy, DNA adducts, strand breaks, and oxidative damage of sperm, decrease men’s fertility [23, 24], and further contribute to PTB.

In our study, we firstly have confirmed that paternal smoking was associated with increased PTB; and we also have identified that exposure to paternal smoking before conception could also independently increase the risk of PTB. In addition to the significant association between paternal smoking and PTB, effects of pre-pregnancy paternal smoking on the risk of PTB were also identified as significant in our study. Compared with women without preconception paternal smoking, the risk of PTB increased, which was consistent with a few previous studies where they found that pre-pregnancy factors such as smoking remained significantly associated with higher risk of preterm delivery [25]. It is suggested that future intervention should therefore be found to reduce HR of PTB by starting before pregnancy.

The current studies were drawn from a large-scale cohort study in over 5 million couples, which ensured the statistical power of analysis, and further permitted consideration of important potential confounders omitted in other analyses. Another strength was that it was the first study that investigated the effects of paternal preconception smoking on hazard risk of PTB. The findings also support the need for further research into effective prevention and intervention strategies specifically for women and their partners who smoke before pregnancy.

Several limitations of the present analysis should be in concern. First, the self-reported smoking frequency may underestimate the true consumption. Expectant mothers and their partners may underreport actual smoking frequency and dose if they perceive smoking during pregnancy as deviant behavior, which could understate effects of smoking frequency or even obscure an association between smoking and PTB, thereby introducing some non-differential misclassification bias. Besides, there may be other lifestyle or environmental factors that we have not taken into consideration which may confound the results. Fathers may have chosen to not smoke in the presence of their pregnant wives and smoke elsewhere instead. Other tobacco exposure sources of women may also be one of the confounding factors.

## Conclusion

We found a significant association between paternal smoking and PTB. The smoking reduction should not only be advised to pregnant women but to their partners to reduce PTB in their fetal. In addition, supporting patients to continue smoking reduction will be crucial when considering the adverse health outcome of smoking. Intervention of tobacco use before and during pregnancy, is critical for the prevention of PTB. Avoiding both maternal and paternal smoking during pregnancy will benefit the developing fetus.

## Supplementary Information

**Additional file 1: Table S1**. Comparison of baseline characteristics between included and excluded participant.

**Additional file 2: Figure S1.** A flow figure for design of the NFPCP.

## Data Availability

NFPCP data contained sensitive data and cannot shared via public deposition because of information governance restrictions in place to protect individuals’ confidentiality. Access to data for external researchers (not affiliated with National Research Institute for Family Planning) requires researchers to be physically based in the institute. Access to data is available only once approval has been obtained through the individual constituent entities controlling access to the data.

## Abbreviations

PTB: Preterm birth
NFPCP: National Free Pre-Pregnancy Checkups Project
BMI: : Body mass index
OR: Odd ratio
HR: Hazard ratio
CI: Confident intervals
RCS: Restricted cubic spline
LMP: Last menstrual period
TSH: Thyroid stimulating hormone
