# Supplementary material for: Paternal smoking and preterm birth: a population-based retrospective cohort study among non-smoking women aged 20–49 years in rural China
Source: Reprod Health. 2022 Mar 24;19:72. doi: 10.1186/s12978-022-01378-x (PMC8944082; doi:10.1186/s12978-022-01378-x)
Supplement: Supplementary file 1 — Additional file 1: Table S1. Comparison of baseline characteristics between included and excluded participant. [file 12978_2022_1378_MOESM1_ESM.pdf]

Supplementary Table 1. comparison of baseline characteristics between included and excluded participants.

Characteristics	Included (N=5,298,043)	Excluded (N=769,624)	P-value
Maternal age at LMP, years	25.20±3.78	25.79±4.21	<0.001
Paternal age at LMP, years	26.93±5.58	27.58±4.84	<0.001
Higher education	656,756 (12.23)	77,194 (11.08)	<0.001
Han ethnic	4,926,310 (91.72)	636,228 (91.31)	<0.001
BMI, km/m^2	21.19±2.85	21.37±3.00	<0.001
Alcohol drinking, ml/day	0.02±0.15	0.03±0.18	<0.001
TSH, mIU/L	1.98±2.46	2.06±2.77	<0.001
Diabetes	62,296 (1.16)	9,577 (1.37)	<0.001
Hypertension	78,613 (1.46)	12,287 (1.76)	<0.001
Primipara	3,696,031 (68.82)	415,671 (59.66)	<0.001
History of adverse pregnancy outcome	166,626 (3.10)	26,226 (3.76)	<0.001
Paternal smoking	3.09±5.94	3.62±6.43	<0.001
Maternal passive smoking, minutes/day	0.88±8.30	1.31±10.21	<0.001
Paternal passive smoking, minutes/day	2.48±14.42	2.90±15.61	<0.001

Abbreviations: BMI: body mass index; LMP: last menstrual period; TSH: thyroid stimulating hormone.
