# Supplementary figures and images for: Paternal smoking and preterm birth: a population-based retrospective cohort study among non-smoking women aged 20–49 years in rural China
Source: Reprod Health. 2022 Mar 24;19:72. doi: 10.1186/s12978-022-01378-x (PMC8944082; doi:10.1186/s12978-022-01378-x)

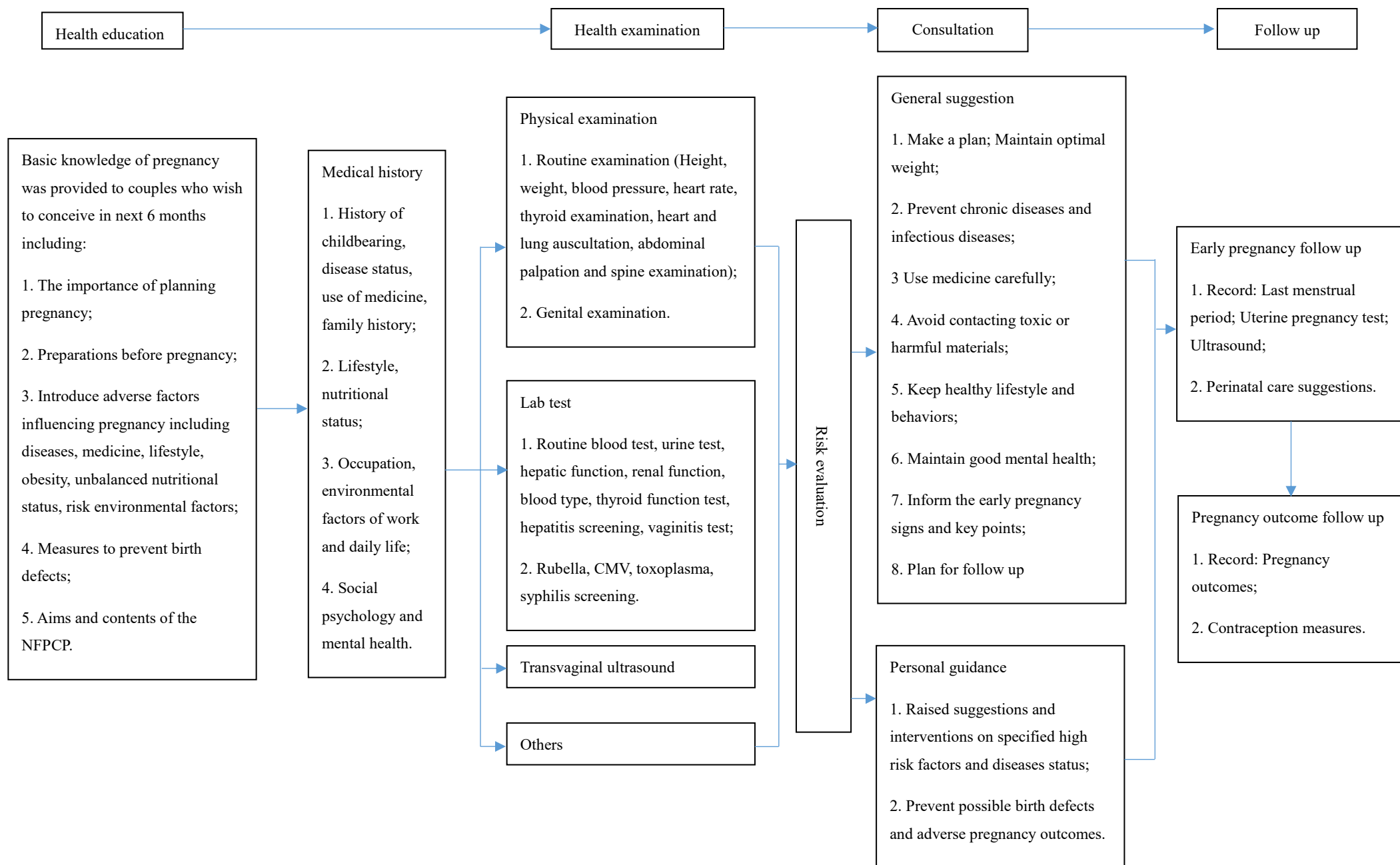

Supplement: Supplementary file 2 — Additional file 2: Figure S1. A flow figure for design of the NFPCP. [file 12978_2022_1378_MOESM2_ESM.pdf]
